# CohereNet: A Deep Learning Architecture for Ultrasound Spatial Correlation Estimation and Coherence-Based Beamforming

**DOI:** 10.1109/TUFFC.2020.2982848

**Published:** 2020-11-24

**Authors:** Alycen Wiacek, Eduardo González, Muyinatu A. Lediju Bell

**Affiliations:** Department of Electrical and Computer Engineering, Johns Hopkins University, Baltimore, MD 21218 USA; Department of Biomedical Engineering, Johns Hopkins University, Baltimore, MD 21218 USA.; Department of Electrical and Computer Engineering, Johns Hopkins University, Baltimore, MD 21218 USA, also with the Department of Biomedical Engineering, Johns Hopkins University, Baltimore, MD 21218 USA, and also with the Department of Computer Science, Johns Hopkins University, Baltimore, MD 21218 USA.

**Keywords:** Coherence-based beamforming, deep learning, spatial correlation, ultrasound

## Abstract

Deep fully connected networks are often considered “universal approximators” that are capable of learning any function. Inthisarticle, we utilize this particular property of deep neural networks (DNNs) to estimate normalized cross correlation as a function of spatial lag (i.e., spatial coherence functions) for applications in coherence-based beamforming, specifically short-lag spatial coherence (SLSC) beamforming. We detail the composition, assess the performance, and evaluate the computational efficiency of CohereNet, our custom fully connected DNN, which was trained to estimate the spatial coherence functions of *in vivo* breast data from 18 unique patients. CohereNet performance was evaluated on *in vivo* breast data from three additional patients who were not included during training, as well as data from *in vivo* liver and tissue mimicking phantoms scanned with a variety of ultrasound transducer array geometries and two different ultrasound systems. The mean correlation between the SLSC images computed on a central processing unit (CPU) and the corresponding DNN SLSC images created with CohereNet was 0.93 across the entire test set. The DNN SLSC approach was up to 3.4 times faster than the CPU SLSC approach, with similar computational speed, less variability in computational times, and improved image quality compared with a graphical processing unit (GPU)-based SLSC approach. These results are promising for the application of deep learning to estimate correlation functions derived from ultrasound data in multiple areas of ultrasound imaging and beamforming (e.g., speckle tracking, elastography, and blood flow estimation), possibly replacing GPU-based approaches in low-power, remote, and synchronization-dependent applications.

## I. Introduction

DEEP learning has achieved state-of-the-art performance for many imaging tasks, including object detection, image segmentation, and image formation. In the field of ultrasonic imaging, this success has resulted in many groups studying how deep learning can replace the well-known, physics-based process of beamforming. Beamforming relies on knowledge of the speed of sound in tissue in order to accurately reconstruct an image from raw channel data. As an alternative to applying physics-based models that assume specific values of this critical speed-of-sound property, recent approaches [1]–[5] use simulated data that incorporate these basic physical principles during training in order to replace the mathematical component of image formation with deep neural networks (DNNs) that learn parameters governing speed-of-sound changes, aberration correction, and other information needed for standard amplitude-based beamforming algorithms (e.g., delay-and-sum (DAS) beamforming).

In particular, when studying the beamforming process from a robotic tracking perspective, Nair *et al.* [1] used plane wave images to produce segmentation maps directly from the RF channel data, bypassing the beamforming step altogether. Allman *et al.* [2] demonstrated the ability to identify true sources and reflection artifacts in photoacoustic images using the Faster R-CNN network configuration, generating high-contrast, artifact-free, high-resolution images of point-like sources by segmenting and displaying only the identified sources with potential applications to ultrasound imaging. Hyun *et al.* [4] demonstrated speckle reduction using a custom CNN, aiming to preserve resolution in comparison to state-of-the-art speckle reduction techniques. Luijten *et al.* [6] presented a deep learning approach to minimum variance beamforming, where ideal weights were learned and applied during the summation step. Additional ultrasound-related deep learning approaches were summarized by van Sloun *et al.* [7].

Although these and other methods have proven to be successful with generating amplitude-based ultrasound (or photoacoustic) images with improved image quality metrics, there are additional advanced imaging methods that can be explored with deep learning approaches. Many of these advanced methods rely on fundamental cross-correlation measurements that can be time consuming to compute. For example, elastography relies on temporal correlations between subsequent frames following the application of an external force [8]. Speckle tracking allows clinicians to estimate cardiac function by tracking ventricular motion through temporal correlations [9]. Doppler imaging uses fundamental cross correlations to compute displacement vectors and calculate blood flow [10]. Recent speed-of-sound estimation approaches [11], [12] rely on the fundamental van Cittert–Zernike theorem applied to ultrasound imaging [13], which requires the calculation of multiple spatial correlation functions (also known as spatial coherence functions). In addition, advanced beamforming algorithms such as the generalized coherence factor [14] and minimum variance beamforming [15] use fundamental cross-correlation measurements to improve contrast and resolution, respectively. As a result, one possible application of deep learning is to remove this critical bottle neck and learn correlation functions, thereby bypassing the repeated correlation calculations that are required for these techniques.

We previously introduced the CohereNet DNN architecture for learning spatial coherence functions [16] with applications to short-lag spatial coherence (SLSC) beamforming [17]. SLSC beamforming is an advanced technique that is based on the fundamental van Cittert–Zernike theorem applied to backscattered ultrasound echo data [13] and therefore relies on the computation of multiple spatial coherence functions, which requires multiple cross-correlation calculations. Specifically, SLSC images are formed by calculating and then summing the spatial coherence of backscattered pressure waves received across the ultrasound transducer, demonstrating improvements over traditional ultrasound image quality in a variety of *in vivo* imaging applications, including breast [18], [19], liver [20], [21], fetal [22], [23], cardiac [24], [25], and thyroid [17] imaging. Benefits of SLSC images include improved visualization of anechoic targets of interest, including breast [18], [19] and thyroid cysts [17] as well as improved endocardial border detection [24], [25]. However, clinical implementation was initially limited by the computational expense associated with multiple repeated correlation calculations.

A graphical processing unit (GPU) SLSC approach was recently introduced to enable real-time clinical implementation [26]. This GPU implementation relies on simplifications, which provide approximations to the underlying spatial correlation. For example, the GPU implementation does not average over an axial kernel to compute the cross correlation and instead includes the kernel dependence after the cross-correlation computations are complete. In the original SLSC algorithm (which we refer to as central processing unit (CPU) SLSC), averaging over an axial kernel enables us to include multiple observations across the aperture, which adds robustness to the cross-correlation estimation.

Therefore, two outstanding challenges for clinical implementation include expected deviations relative to the original CPU SLSC algorithm proposed by Lediju *et al.* [17] and the speed of delivering real-time results that are faithful to the original SLSC algorithm. Deep learning is one possible option to address these two challenges, which is the focus of this manuscript. Specifically, we expand on the work presented in our associated conference paper [16] to summarize the technical details of CohereNet, further developing and deploying it to bypass the repeated correlation calculations needed to otherwise form SLSC images, without requiring the mathematical simplifications described in [26]. We explore this approach considering the “universal approximation” property of DNNs, relying on this particular property to estimate spatial coherence functions and create SLSC images. We then investigate the similarity of the resulting images relative to the originally proposed CPU SLSC algorithm and the GPU simplification approach.

The remainder of this article is organized as follows. Section II provides an overview of the SLSC algorithm, describes its mathematical relationship to CohereNet input data, details the methods used to generate training data, and describes the custom CohereNet architecture built for the proposed task of learning spatial coherence functions. Section III demonstrates the performance and computational efficiency of CohereNet, using a variety of *in vivo* breast lesions, as well as data from tissue-mimicking phantoms, and *in vivo* liver. Section IV discusses our findings and their implications for other areas of ultrasound imaging. Finally, Section V summarizes the major contributions of this article.

## II. Methods

### A. SLSC Beamforming

**Fig. 1.**
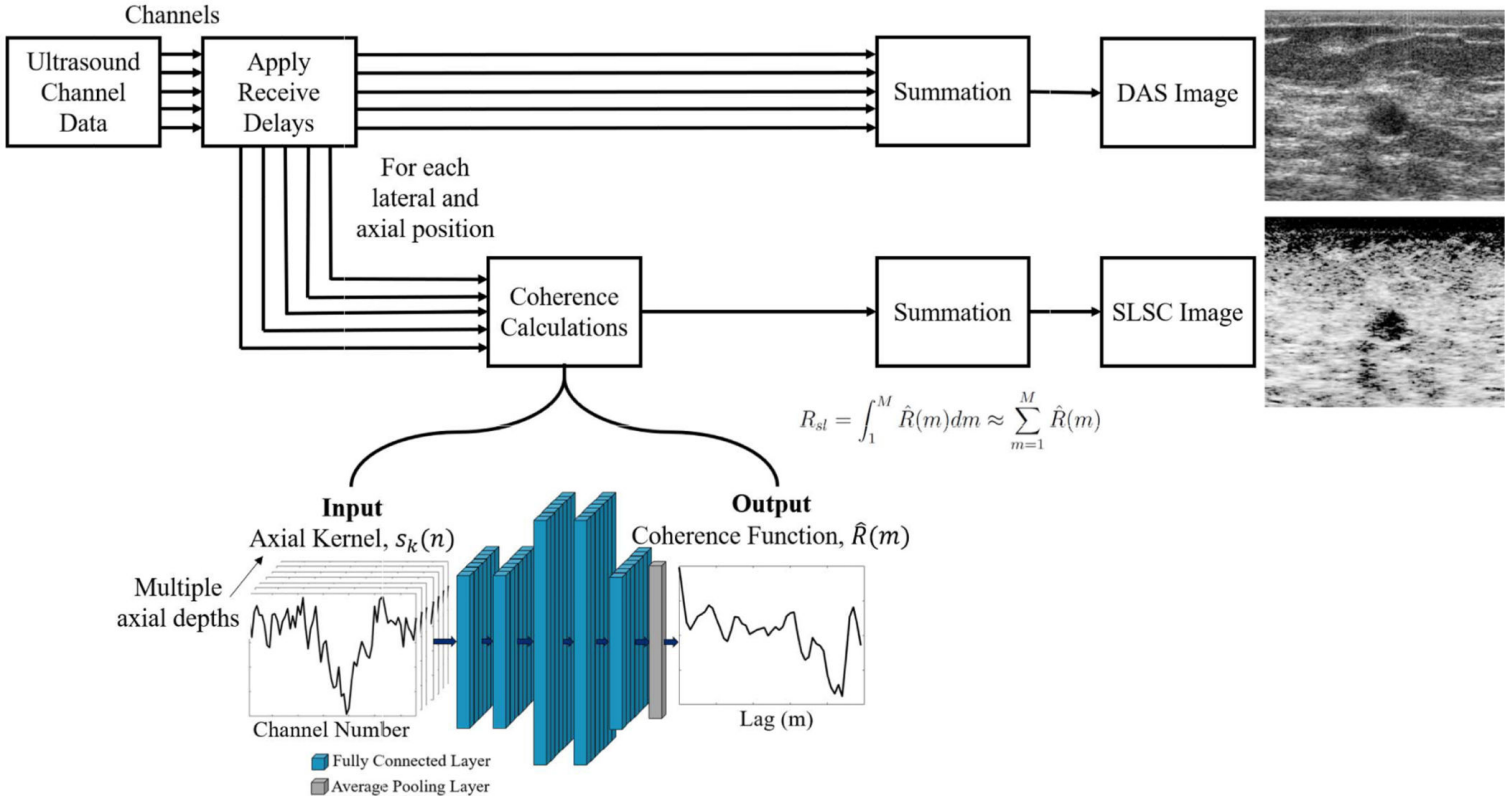
Process flow diagram comparing DAS beamforming to SLSC beamforming and demonstrating where CohereNet is implemented in the SLSC image formation pipeline.

SLSC beamforming calculates and directly displays the spatial coherence of backscattered ultrasound pressure waves received across an array of ultrasound transducer elements. This approach can be contrasted with traditional DAS beamforming, which provides images of recorded pressure amplitude (as illustrated in Fig. 1). To implement SLSC beamforming, after standard receive delays are applied, normalized correlation measurements are calculated between equally spaced elements, or lags, resulting in the normalized spatial correlation, defined as

(1) $$\hat{R}(m)=\frac{1}{N-m}\overset{N-m}{\underset{i=1}{\sum }}\frac{\sum_{k}s_{k,i}(n)s_{k,i+m}(n)}{\sqrt{\sum_{k}s_{k,i}^{2}(n)\sum_{k}s_{k,i+m}^{2}(n)}}$$

where *m* is the lag in the number of elements, *N* is the number of receive elements in the transducer, and *s*_*k,i*_*(n)* is a time-delayed, zero-mean kernel consisting of *k* axial samples, each received at element *i* and centered at depth *n*.

The resulting spatial coherence function, $\hat{R}(m)$, is then summed up to a specific short-lag value, *M*, yielding the value of the SLSC pixel

(2) $$R_{sl}=\overset{M}{\underset{1}{\int }}\hat{R}(m)dm\approx \overset{M}{\underset{m=1}{\sum }}\hat{R}(m).$$

This process was repeated for each lateral and axial positions in the image, with an axial correlation kernel length of approximately one wavelength, based on previous investigations of optimal kernel lengths [27]. One wavelength ranges from 3 to 13 axial samples for transmit frequencies in our data set. We selected a fixed kernel length near the midpoint of these values (i.e., seven axial samples) for our implementations throughout this article.

The axial kernel of channel data is related to the CohereNet architecture as follows:

(3) $$s_{k}(n)=\left\{s_{k,i}(n)\forall i=1,\ldots ,N\right\}$$

which states that *s*_*k*_*(n)* is equivalent to *s*_*k,i*_ *(n)* when all channels from *i* = 1 to *i* = *N* are stacked side-by-side and ordered from 1 to *N*. Equation (3) is a mathematical representation describing one axial kernel of ultrasound channel data consisting of *k* axial samples, each measured across the entire receive aperture and centered at depth *n*.

In contrast, the single sample ensemble GPU implementation does not use an axial kernel to compute the cross correlation [i.e., *k* in (1)] and computes each cross correlation in the numerator of (1) separately from the normalization in the denominator. These modifications result in a general normalization factor applied to multiple values at a particular lag, instead of each value being normalized individually, as in CPU SLSC [26].

### B. Training, Testing, and Validation Data Sets

A data set was generated using *in vivo* breast ultrasound data from 24 different patients, obtained after informed consent and approval from the Johns Hopkins Medicine Institutional Review Board [18], [19]. Data were acquired with an Alpinion ECUBE-12R research ultrasound scanner connected to an Alpinion L8–17 linear array ultrasound transducer (Alpinion, Seoul, South Korea). The transducer has 128 elements, with 64 elements allowed to receive at one time (i.e., *N* = 64).

The acquired data were split into training, validation, and testing sets by patient. A total of 18 patients were included during training, three additional patients were used for validation, and three additional patients were used for testing. The data from each patient consist of two orthogonal scans (i.e., radial and anti-radial), each with 10 frames/scan. One training example is defined as one axial kernel of channel data, that is, *s*_*k*_*(n)*, and its corresponding coherence function, that is, $\hat{R}(m)$. Considering that there were approximately 2000 axial kernels/scan line, a total of 92.2 million examples were included in the training data set. Similarly, 15.4 million examples were included in either the validation or test data sets.

To test generalizability, additional test frames were acquired using three additional ultrasound transducers, each with 64 receive elements and a minimum of 128 scanlines, with transmit focal depths ranging from 7 mm to 6.9 cm: 1) an Alpinion SP1–5 phased array transducer; 2) an Alpinion EC3–10 curvilinear array transducer; and 3) a Verasonics P4–2v phased array attached to a Verasonics research-based ultrasound system (Verasonics, Kirkland, WA). Each of the three additional transducers were used to acquire images of a CIRS Model 054GS phantom (CIRS, Norfolk, VA). In addition, an *in vivo* liver data set acquired with an Alpinion L3–8 linear array transducer [20] was also included during testing.

To acquire volumetric data with multiple anechoic regions of interest (ROIs) for SLSC imaging, data from a CIRS Model 050 small parts phantom was acquired with an Alpinion L3–8 linear array transducer, while scanning in an L-shaped motion, moving from the 5-mm anechoic cyts, to the 3-mm anechoic cysts, and over to the 10-mm anechoic cyst and point targets.

### C. Network Input Data

In order to build a data set that contained enough valid measurements of coherence, the acquired raw data were first delayed and filtered prior to network input. Due to the sliding window of selected active receive subapertures of the Alpinion L8–17 linear array, the training data were filtered to include only the center scanlines, which ensured not less than 64 receive elements for each computation. The coherence functions were then computed (using an axial kernel length of seven samples, as noted in Section II-A). The first and last three axial SLSC image pixels of each acquisition were ignored, in order to avoid introducing errors due to coherence measurements over an incomplete kernel of data.

### D. CohereNet Architecture

In order to model (1), a custom DNN architecture was implemented using Keras [28] with a Tensorflow backend [29]. The input to the network is represented as $s_{k}(n)\in {\mathbb{R}}^{k\times N}$, and the output is $\hat{R}(m)\in {\mathbb{R}}^{1\times m}$, where *m* is the number of lags to be computed. Modeled after the mathematical cross-correlation function, the network comprises of an input layer followed by four fully connected layers and an average pooling layer as shown in Table I. A rectified linear unit (ReLU) activation function was used for the first three fully connected layers due to its promotion of sparsity in the activation map and ability to avoid vanishing gradients [30]. A hyperbolic tangent (*tanh*) activation function followed on the final fully connected layer in order to limit the output of the network between −1 and 1, similar to the mathematical cross-correlation function. The dependence on an axial kernel is retained throughout the network until the final layer. We call this network architecture CohereNet. CohereNet contains 37248 parameters and has a memory requirement of 149 kB (when using a 32-bit floating point value to store each parameter).

CohereNet was trained using a modified mean squared error (MSE) loss function, defined as

(4) $$\text{MSE}=\frac{1}{M}\overset{M}{\underset{m=1}{\sum }}w_{m}{\left(y_{m}-{\hat{y}}_{m}\right)}^{2}$$

where *m* is the lag, *y*_*m*_ is the computed coherence function, ${\hat{y}}_{m}$ is the ground truth coherence function, and *w* is a vector of Gaussian weights with *μ* = 0 and *σ* = 25.6. This custom Gaussian weighting scheme was used to place larger weight on errors in the short-lag region (i.e., the region used to create SLSC images, and therefore the region most critical to improving SLSC image quality).

CohereNet was trained using empirically optimized hyperparameters including: a batch size of 128, an Adam optimizer [31], five epochs, and a learning rate of 0.001. The PC used for training was an Intel Core i5–6600k CPU with 32 GB of RAM alongside an Nvidia GTX Titan X (Pascal) with 12 GB of VRAM and a core clock speed of 1531 MHz.

### E. Computational Speed and Complexity

In order to compare previous implementations of SLSC with CohereNet, the same channel data were processed on the same computer (i.e., the PC described in Section II-D) for each implementation. The three implementations for comparison were: 1) the original SLSC algorithm, implemented on a CPU with MATLAB mex functions (i.e., CPU SLSC); 2) GPU SLSC, implemented on a GPU with mathematical simplifications to the spatial coherence function summarized in Section II-A and described in [26]; and 3) DNN SLSC, which utilizes CohereNet and was implemented on a GPU with Tensorflow and Keras.

Computational speed was measured over ten iterations of the same image. In addition, speed was reported as a function of resampled image sizes prior to calculations. For this image size comparison, images were both upsampled by a factor of 2 and downsampled by factors of 2, 4, and 8.

In addition, the number of floating point operations (FLOPs) required to create GPU SLSC and DNN SLSC images were calculated to compare the computational cost of each approach. For GPU SLSC, the number of FLOPs was measured using the CUDA Nsight tool and Visual Studio 2017. For DNN SLSC, because the Nsight estimation tool is not available for Keras, we calculated the number of FLOPs manually by counting the matrix multiplication and vector additions required for one forward pass through the network [6]. Each fully connected layer requires 2*N*_*i*_ *N*_*i*+_1 + *N*_*i*+_1 FLOPs, where *N*_*i*_ and *N*_*i*+1_ are the number of input and output nodes, respectively, within each layer *i* [32]. ReLU requires a comparison and a multiplication, therefore it requires two FLOPs, *tanh* requires five multiplications and three additions, therefore it requires eight FLOPs [33], and average pool requires one multiplication and six additions, therefore it requires seven FLOPs. The overall number of FLOPs required for one forward pass through the network, resulting in the network output $\hat{R}(m)$, can be represented by

(5) $$O_{\hat{R}}=k\left[\sum_{i=0}^{L-2}\underset{\text{FC}\;\text{layer}\;}{\underset{︸}{(2N_{i}N_{i+1}+N_{i+1}}}+\underset{\text{activation}\;}{\underset{︸}{2N_{i+1})}}\;+\;\underset{\text{last}\;\text{FC}\;\text{layer}}{\underset{︸}{2N_{L-1}N_{L}+N_{L}}}+\underset{\text{activation}}{\underset{︸}{8N_{L}}}+\underset{\text{avg}.\;\text{pool}}{\underset{︸}{7N_{L}}}\right]$$

where *N*_*L*−1_ and *N*_*L*_ are the number of input and output nodes, respectively, within the last layer, *L* = 4, and *k* = 7 as defined in Section II-A. Fully connected is abbreviated as FC in (5). The total number of FLOPs to form each SLSC image was determined by multiplying $O_{\hat{R}}$ by the total number of axial × lateral samples in the image.

### F. Image Quality Metrics

The quality of images were quantitatively compared by computing the contrast, signal-to-noise ratio (SNR), contrast-to-noise ratio (CNR), and generalized CNR (gCNR) [34], [35] of matched CPU SLSC and DNN SLSC images created from the same channel data, defined as follows:

(6) $$\text{Contrast}=20\log_{10}\left(\frac{S_{i}}{S_{o}}\right)$$

(7) $$\text{SNR}=\frac{S_{o}}{\sigma_{o}}$$

(8) $$\text{CNR}=\frac{\left|S_{i}-S_{o}\right|}{\sqrt{\sigma_{i}^{2}+\sigma_{o}^{2}}}$$

(9) $$\text{gCNR}=1-\overset{1}{\underset{x=0}{\sum }}\underset{x}{\min}\left\{p_{i}(x),p_{o}(x)\right\}$$

where *S*_*i*_ and *σ*_*i*_ are the mean and standard deviation, respectively, within an ROI inside of the target, *S*_*o*_ and *σ*_*o*_ are the mean and standard deviation, respectively, within an ROI outside of the target (both computed prior to log-compression), and *p*_*i*_ and *p*_*o*_ are the probability density functions of the signal inside and outside the target, respectively.

For each of the *in vivo* breast masses in the test set, the inside ROI was selected from inside the mass, and the outside ROI was selected at the same axial depth and size outside of the mass wherever possible. For the phantom and *in vivo* liver data sets, the inside ROI was inside the most anechoic or hypoechoic region, with a corresponding outside ROI at the same axial depth within the tissue portion of the phantom or liver. All images were normalized to the brightest pixel within the image and displayed on a linear scale with a maximum normalized brightness of 1 and a minimum of 0.

In addition, the correlation between each CPU SLSC image and its corresponding GPU SLSC or DNN SLSC image created from the same channel data was computed for these matched image pairs in order to provide a more global measure of similarity that is not dependent on ROI selection.

## III. Results

### A. In Vivo Breast Test Set

**Fig. 2.**
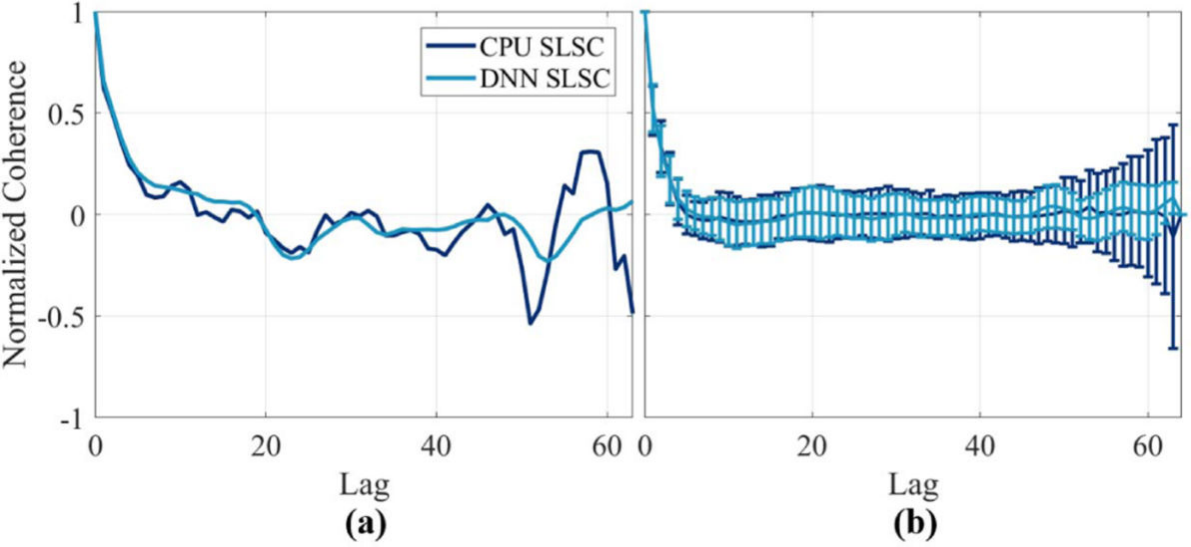
(a) One example coherence function from the focus of *in vivo* breast data. (b) Mean ± standard deviation coherence function of 1 mm × 1 mm region surrounding the focus of *in vivo* breast data.

Fig. 2(a) shows an example coherence function measured at the focus of one of the images in the test set of *in vivo* breast data. The DNN result appears to fit the CPU result better at low lags (i.e., ≤25) compared with higher lags (i.e., >25), which is expected given the weighted loss function in (4). Although the MSE difference between these two functions is 0.02, when considering the region where penalties for mismatch was larger (i.e., lags ≤25), the MSE is 0.002. Otherwise, for lags >25, the MSE is 0.03.

Fig. 2(b) shows the mean ± standard deviation of coherence functions within a 1 mm × 1 mm region surrounding the focus of the data set used to make Fig. 2(a). While the DNN result in Fig. 2(a) seems to smooth the coherence function in comparison to the CPU result, we observed other cases where CohereNet produced results that more closely followed the CPU-generated coherence functions in the short-lag region. Therefore, the results in Fig. 2(b) are a better representation of the average match that we observed. These results further emphasize the trend of better matches between CPU- and DNN-generated coherence functions in the short-lag region (compared with results obtained outside of the short-lag region).

**Fig. 3.**
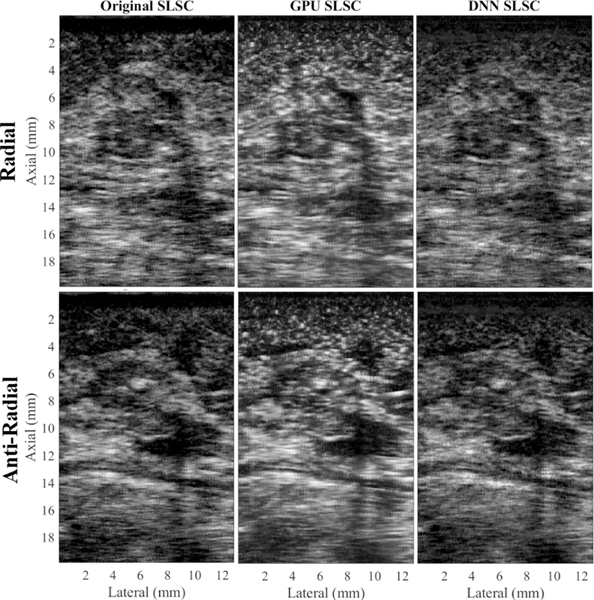
Example images from the test set containing *in vivo* breast data, showing radial (top) and anti-radial (bottom) views. Each image triplet was generated using CPU (left), GPU (center), and DNN (right) SLSC images, created from the same channel data. All images were normalized and displayed on a linear scale from 0 to 1.

Fig. 3 shows two triplets of matched CPU, GPU, and DNN SLSC images of two orthogonal (i.e., radial and anti-radial) acquisitions in the test set of one *in vivo* breast mass from one patient. The radial and anti-radial scans of this breast mass are shown in the top and bottom rows of Fig. 3, respectively. Each triplet shows CPU, GPU, and DNN SLSC images, from left to right, respectively. Qualitatively, within each triplet, the DNN SLSC image looks more similar to the CPU SLSC image than the GPU SLSC image. Each image is normalized and displayed from 0 to 1. The GPU SLSC image appears smoother and brighter, with higher contrast than the CPU SLSC image. Quantitatively, over the entire test set of *in vivo* breast data, the mean correlation between CPU SLSC and DNN SLSC images was 0.93, which highlights the observed similarity between the CPU SLSC images and the DNN SLSC images. In comparison, the mean correlation between CPU and GPU SLSC images was lower at 0.86.

**Fig. 4.**
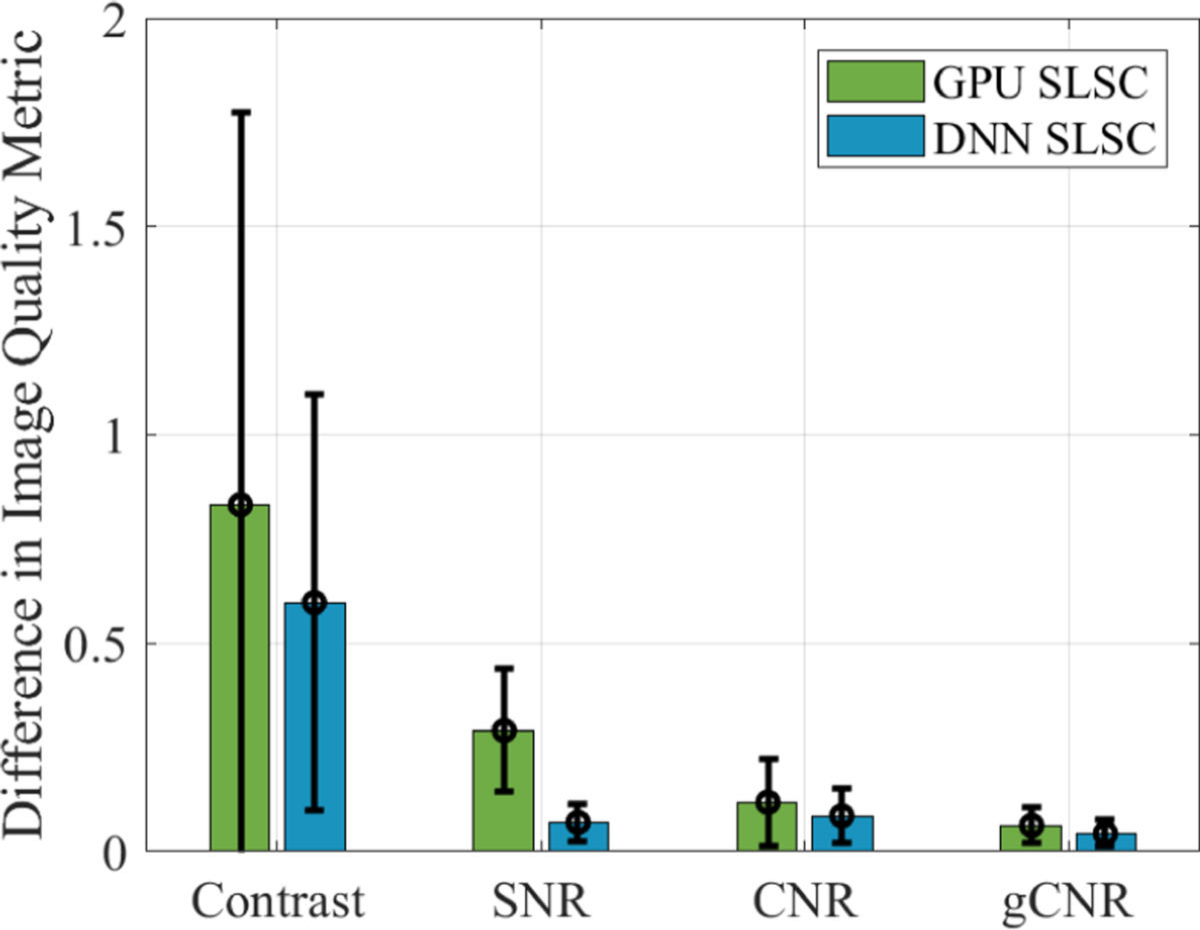
Differences in contrast, SNR, CNR, and gCNR when comparing CPU SLSC images to GPU or DNN SLSC images. The mean is shown as a bar, with ± one standard deviation shown as an error bar on the mean.

The blue bars in Fig. 4 show mean differences in contrast, SNR, CNR, and gCNR between CPU and DNN SLSC images over the entire test set of *in vivo* breast ultrasound data, with error bars representing ± one standard deviation of these difference measurements. Averaged over the entire test set, the mean contrast, SNR, CNR, and gCNR differences were 0.5 dB, 0.1, 0.07, and 0.04, respectively. For comparison, the green bars in Fig. 4 show the mean differences in contrast, SNR, CNR, and gCNR between CPU and GPU SLSC, demonstrating larger mean differences of 0.8 dB, 0.3, 0.1, and 0.06, respectively, and larger standard deviations.

### B. Demonstration of Network Generalizability

**Fig. 5.**
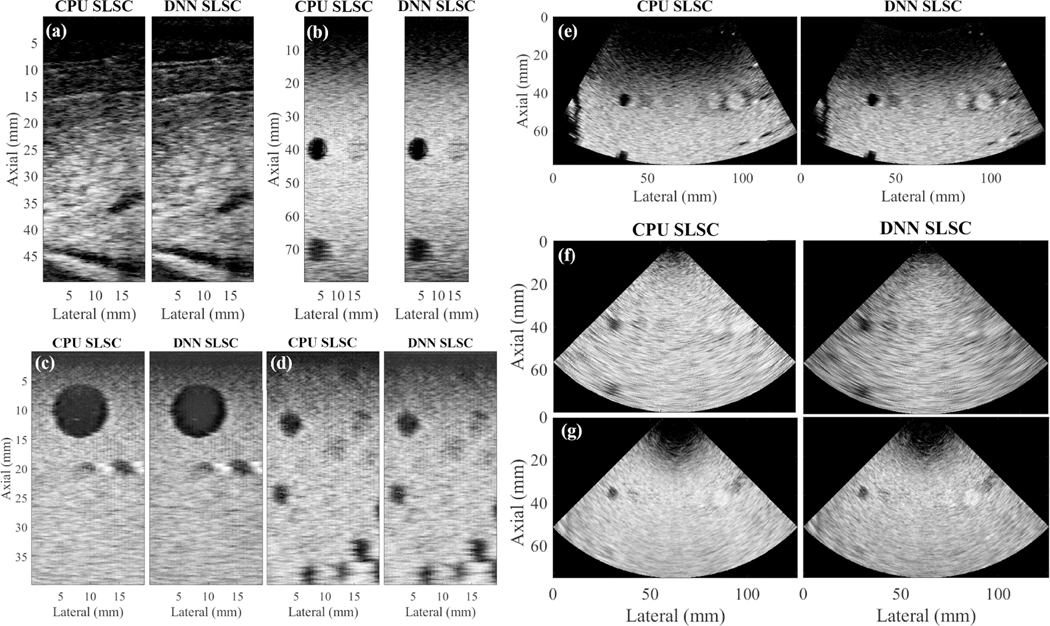
Example pairs of matched CPU and DNN SLSC images from the additional test set acquired with an Alpinion L3–8 linear array to image (a) *in vivo* liver tissue, (b) CIRS Model 054GS phantom, and (c and d) two orthogonal views of a CIRS Model 050 phantom. The CIRS Model 054GS phantom was additionally imaged with (e) Alpinion SC1–6 curvilinear array, (f) Alpinion SP1–5 phased array, and (g) Verasonics P4–2v phased array connected to a Verasonics ultrasound imaging system. All images were normalized and displayed on a linear scale from 0 to 1.

Fig. 5 shows matched pairs of CPU and DNN SLSC images created after testing with *in vivo* liver, multiple probe geometries, and a Verasonics (as opposed to Alpinion) ultrasound system. Specifically, Fig. 5(a) shows the result of applying CohereNet to *in vivo* liver data acquired with the Alpinion L3–8 linear array. Fig. 5(b) shows the results obtained with the CIRS 054GS phantom and the Alpinion L3–8 linear array. Fig. 5(c) and (d) shows the results obtained from two orthogonal planes of the CIRS 050 phantom with the Alpinion L3–8 linear array. Fig. 5(e)–(g) shows phantom results obtained with the Alpinion SC1–6 curvilinear array, the Alpinion SP1–5 phased array, and the Verasonics P4–2v phased array, respectively. A majority of these images have a notable dark-region artifact at the top of the image due to the use of focused transmits [36]. The dark regions to the left and right of the point targets and hyperechoic cyst in Fig. 5(e) are caused by the high-amplitude signals from these targets generating high-amplitude off-axis scattering lateral to the targets [37].

When averaged over the entire test set of phantom and *in vivo* liver data, the mean contrast, SNR, CNR, and gCNR are similar (i.e., ≤12% difference) when comparing CPU SLSC images to DNN SLSC images. In addition, over the entire test of phantom and *in vivo* liver data, the mean correlation between CPU SLSC and corresponding DNN SLSC images was 0.96. When the *in vivo* breast test data set was included, the mean correlation over the entire test set of phantom, *in vivo* liver, and *in vivo* breast data was 0.93. A summary of the image-to-image correlation for each test set appears in Table II.

### C. Computational Comparisons

**Fig. 6.**
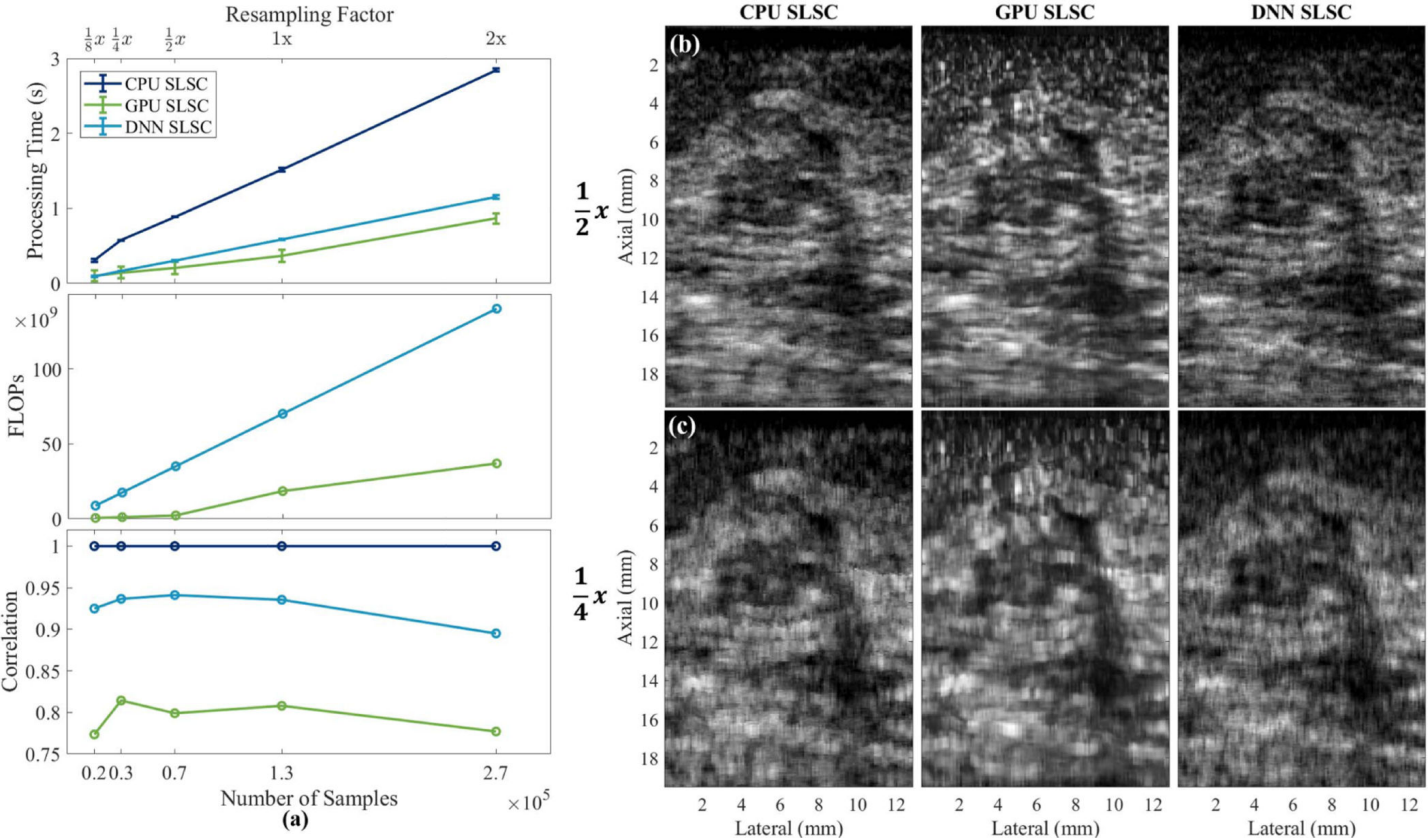
(a) Processing times, FLOPs, and image-to-image correlations as functions of the number of samples (i.e., lateral × axial samples) included in each SLSC implementation (i.e., CPU, GPU, or DNN SLSC). Example images created with resampling factors of (b) 1/2 and (c) 1/4.

Fig. 6(a) shows computation times, FLOPs, and image-to-image correlations (from top to bottom, respectively) plotted against the number of axial × lateral samples shown in the radial view of Fig. 3, after applying resampling factors ranging from 1/8 to 2. The standard deviations for the processing times are shown as error bars on the mean. No standard deviations are reported for the FLOPs or the correlation results because the processing times were computed with the exact same data run through each algorithm ten times (i.e., ten iterations), and each iteration returned identical values per resampling factor.

The computation times shown in Fig. 6(a) improve rapidly as the number of samples decreases for CPU SLSC. Both GPU SLSC and DNN SLSC have similarly improved computational speed as the number of samples decreases. With 128 scanlines and 130 axial samples (i.e., 0.2 × 10^5^ samples), the minimum processing times for CPU, GPU, and DNN SLSC in Fig. 6(a) are 0.31, 0.09, and 0.09 s, respectively. These processing times correspond to frame rates of 3, 11, and 11 Hz, respectively.

The corresponding FLOP results in Fig. 6(a) suggest that DNN SLSC requires more FLOPs than GPU SLSC for each resampling factor. However, the increased number of FLOPs does not directly translate to an increase in processing time, considering that the processing times for GPU SLSC and DNN SLSC with ≤0.7 × 10^5^ samples are comparable. Although FLOPs are representative of computational complexity, processing time is a better representation of computational speed because SLSC is ultimately intended to be a real-time imaging method.

The corresponding image-to-image correlation results shown in Fig. 6(a) demonstrate a degradation in image quality as the number of axial samples decreases. Note that the correlation for CPU SLSC is consistently equal to one because it is correlated with itself, and each CPU SLSC image is the independent baseline for the corresponding DNN and GPU images created with the same resampling factor in order to correlate images containing the same number of samples. Over all of the sampling schemes, the DNN SLSC image has higher correlation with the CPU SLSC image than the GPU SLSC image.

To provide example images of the DNN SLSC images that were created with similar processing times to those of GPU SLSC images, Fig. 6(b) and (c) show images downsampled by factors of 1/2 and 1/4, respectively. These images can be compared with their original versions appearing in the top of Fig. 3 which have a resampling factor of 1 (i.e., no resampling). The downsampled images demonstrate the decreased resolution that is responsible for the decreased correlations observed in the bottom of Fig. 6(a). Although the processing times for the 1/4 resampling results are similar between GPU and DNN SLSC images, the degraded resolution observed in Fig. 6(c) can render this image as unacceptable. At the next highest resolution evaluated (i.e., 1/2 resampling factor), Fig. 6(a) demonstrates that the mean processing time for the DNN SLSC image is within one standard deviation of the processing times for GPU SLSC images, while producing a mean image-to-image correlation similar to that achieved with higher resolution DNN images. Qualitatively, the images in Fig. 6(b) also appear to be similar to the original images displayed in Fig. 3(a).

## IV. Discussion

### A. Advantages of DNN Approach to Correlation Calculations

There are two main advantages to using a DNN to estimate spatial coherence functions. First, the DNN enables bypassing of the repeated and time-intensive mathematical correlations calculation step, providing speedup by a factor of 3.4 when compared with the CPU SLSC approach. It is also remarkable that the network is sufficiently generalizable across two ultrasound system manufacturers, multiple data types, and multiple ultrasound transducer array geometries. Because the network was trained with *in vivo* breast data, which is known to be highly heterogeneous, we hypothesize that multiple variations in coherence functions from this training data set provided sufficient variations for the network to learn multiple examples of coherence functions. As a result, the network was able to generalize to multiple unseen cases. For example, Fig. 5(f) and (g) show the same phantom imaged using an Alpinion phased array and a Verasonics phased array. Although these channel data were generated using two different ultrasound imaging systems, the network nonetheless accurately estimated coherence regardless of the differing ultrasound systems.

The second advantage to a DNN-based approach is the reduced estimation error compared with the GPU implementation. The GPU approach uses mathematical simplifications in order to parallelize the mathematical calculations, which also cause estimation errors. In addition, the accuracy and performance of operations performed on the GPU depend on the resolution of the floating-point variables (i.e., the spatial coherence calculated on a CPU uses the double class of variables, whereas the GPU uses floating-point variables). The DNN calculations are performed on the same GPU and are therefore also subject to floating-point errors. However, because the DNN is trained to mimic the coherence function, particularly in short-lag regions, we suspect that the DNN inherently corrects for errors in floating point values, resulting in DNN images that are both quantitatively and qualitatively more similar to the CPU images than their GPU counterparts, as demonstrated in Fig. 3 and quantified in Fig. 4. Outside of the short-lag region (i.e., >25 lags), less weight was placed on DNN estimation errors, resulting in the smoother coherence functions at these lags (see Fig. 2), which is acceptable for SLSC image formation.

While the current study focused on learning the coherence function only, DNN architectures like CohereNet could be extended to learn more complex operations found in other advanced beamforming algorithms, such as R-SLSC [20] or LW-SLSC [38], which may otherwise be challenging or not feasible to implement in parallel on a GPU. These additional applications will be the focus of future work.

### B. Implications for Computationally Efficient Systems

Aside from being the most mathematically accurate, one main advantage of the CPU SLSC approach is the ability to process data on the same CPU where raw data may be stored, thus bypassing the transfer of the raw data to the GPU that is required for any GPU-based approach. However, the advantages of beamforming coherence images in parallel on a CPU are limited and often depend on additional processors or servers, such as the Message Passing Interface workflow [39], which introduces additional memory transfer issues. Therefore, without multiple core processors or servers with a fast data transfer channel, a CPU SLSC implementation cannot be currently applied to real-time imaging applications. On the other hand, the GPU SLSC approach effectively manages the multiple processor architectures. With sufficient samples to overcome the memory transfer requirement, it is advantageous to use some level of estimation to allow parallel processing, which can be achieved with either a GPU or a GPU-based DNN approach.

Fig. 6(a) demonstrates that the GPU and DNN SLSC algorithms both increase processing times as the number of samples increases. However, the standard deviations of processing times are smaller when using DNN SLSC compared with GPU SLSC, which is likely due to the stability of the Tensorflow and Keras GPU packages and efficiency in memory transfer. While downsampling to 1/2 the size of the original image is sufficient to visualize breast mass features (compare Fig. 6(b) with Fig. 3), and the computation times for GPU and DNN SLSC algorithms are similar for this reduced number of samples [see Fig. 6(a)], the lower standard deviation of the DNN approach would be more desirable for applications that require synchronization with consistent and predictable frame rates. In addition, network optimizations are likely possible in order to improve the computation time of DNN SLSC [40].

Another option for fast and computationally efficient implementations are field-programmable gate arrays (FPGAs), which can be used to implement DNNs [41]–[43] with low energy consumption per float-point operation at lower computational cost than GPU implementations [44]–[46]. These low-power implementations would be useful for embedded system applications in remote areas of the world, where high-energy computing is not feasible. An additional potential benefit is low-power DNN-based FPGA implementations of SLSC for miniaturized ultrasound imaging systems. When comparing CohereNet to compact DNNs targeted for use in mobile applications (which have >4 million parameters [47]), our network requires at least 100× less memory than these compact designs. Therefore, we consider CohereNet to have a memory footprint that is sufficiently compact for low-power applications.

### C. Potential Clinical Applications

SLSC has previously been shown to successfully distinguish solid from fluid-filled hypoechoic breast masses [18], which is one possible clinical application of a real-time DNN-based approach to SLSC imaging that is closely aligned with the originally proposed algorithm implemented on a CPU. Other previously demonstrated clinical advantages of SLSC imaging [21]–[26], including an SLSC-based approach to blood flow imaging [48], [49], would also benefit from the CohereNet architecture. In addition, given the demonstrated generilizability of the network, CohereNet is promising for other areas of ultrasound imaging where fundamental cross-correlation calculations are required, including elastography, speckle tracking, sound speed correction, and other advanced beamforming algorithms, such as minimum variance beamforming.

## V. Conclusion

This work is the first to use the universal approximation properties of DNNs in order to estimate spatial coherence functions and create coherence-based SLSC ultrasound images that are both qualitatively and quantitatively similar to their CPU SLSC counterparts. Over the entire test set (which includes *in vivo* breast and liver data), the average correlation between the DNN SLSC image and the matched CPU SLSC image was 0.93, which demonstrates the *in vivo* clinical feasibility of CohereNet. DNN SLSC images were generated with a frame rate as high as 11 fps with 128 scanlines and 130 axial samples, which is comparable to current GPU implementations. In addition, CohereNet was able to generalize across a variety of tissue types, transducer geometries, and ultrasound imaging systems, which is promising for learning the correlation calculations needed for extended applications of correlation-based ultrasound imaging, possibly replacing GPU-based approaches in low-power, remote, miniaturized, and synchronization-dependent applications.

## Figures and Tables

**TABLE I:** CohereNet Architecture

Layer	Type	Size	Activ.
Input	-	7 × 64	-
1	Fully Connected	7 × 64	ReLU
2	Fully Connected	7 × 128	ReLU
3	Fully Connected	7 × 128	ReLU
4	Fully Connected	7 × 64	Tanh
Output	Average Pool	1 × 64	-

**TABLE II:** Summary of Network Generalizability

		Transducer Parameters							
Test Dataset	Transducer Manufacturer Array & Model	Center Frequency (MHz)	Transmit Frequency (MHz)	Sampling Frequency (MHz)	Number of Transmit Elements	Number of Receive Elements	Pitch (mm)	Transmit Focal Depth (mm)	Mean Correlation
		
*In Vivo* Breast	Alpinion Linear L8-17	10.6	12.5–14	40	128	64	0.2	7–12	0.93
*In Vivo* Liver	Alpinion Linear L3-8	5.4	8	40	128	64	0.3	37	0.97
		
054GS/050 Phantoms	Alpinion Linear L3-8	5.4	8	40	128	64	0.3	20–40	0.98
054GS Phantom	Alpinion Phased SP1-5	3	3.5	40	64	64	0.3	44	0.92
054GS Phantom	Verasonics Phased P4-2v	3	3	11.9	64	64	0.3	40	0.99
054GS Phantom	Alpinion Curvilinear SC 1-6	4	3.6	40	128	64	0.5	69	0.97
